# Proteomics of human duodenum in pre-diabetes and type 2 diabetes reveals potential novel therapeutic targets for aetiology and therapeutics

**DOI:** 10.1186/s12014-026-09595-3

**Published:** 2026-03-16

**Authors:** Beatriz D.G. Alves, Paula Monteiro, Pedro C. Martins, José Braga, Rune Matthiesen, José Flávio G. Videira, Inês G. Mollet

**Affiliations:** 1 UCIBIO - Applied Molecular Biosciences Unit, NOVA School of Science and Technology, Universidade NOVA de Lisboa, Caparica, Portugal; 2 i4HB - Institute for Health and Bioeconomy, NOVA School of Science and Technology, Universidade NOVA de Lisboa, Caparica, Portugal; 3 Serviço de Anatomia Patológica, Instituto Português de Oncologia do Porto FG, EPE (IPO-Porto), Porto, Portugal; 4 Clínica de Patologia Digestiva, Instituto Português de Oncologia do Porto FG, EPE (IPO-Porto), Porto, Portugal; 5 iNOVA4Health, NOVA Medical School, Universidade NOVA de Lisboa, Lisbon, Portugal

**Keywords:** Type 2 diabetes, Pre-diabetes, Duodenum, Proteomics, Proteome, Biomarker

## Abstract

**Background:**

Remission from pre-diabetes and type 2 diabetes (T2D) is frequently observed immediately after a duodenal/jejunal bypass. This demonstrates the reversibility of T2D and involvement of the proximal small intestine in T2D pathology. This study investigates the role of the duodenum in the pathophysiology of T2D, in line with the hypothesis, that exclusion of duodenum/jejunum in T2D (for example, in RYGB surgery) prevents release of unidentified factors that impair insulin sensitivity, leading to T2D remission. The study aimed at identifying novel protein therapeutic targets in human duodenum in T2D and clarifying aetiology.

**Methods:**

Proteome analysis using nano LC-MS/MS was performed on human duodenum surgical samples: T2D (*n* = 9), pre-diabetics (*n* = 6) and non-diabetics (*n* = 11). Proteins were quantified and analysed using MaxQuant and Perseus software. Linear discriminant (LDA), repeated k-fold cross-validation and receiver operating characteristic (ROC) analyses were used to segregate disease groups for the highest-ranking proteins. Pilot mRNA expression of five selected protein targets was analysed using real-time RT-qPCR on duodenum from human and the pre-clinical high-fat diet C57BL/6J mouse model of pre-diabetes.

**Results:**

Proteomics identified and quantified significant changes in 23 proteins (one-way-ANOVA and Holm adjusted pairwise comparisons), differentiating pre-diabetes and T2D. LDA analyses accurately distinguished disease states and identified highest ranking for 10 proteins distinguishing type 2 diabetics, pre-diabetics and non-diabetics with first linear discriminant LD1 explaining 92% of differences; of which three proteins increased in pre-diabetes (DYNC1LI1, KIF5B and MAPRE1; ROC AUC = 0.89,0.82 and 0.86) are involved in vesicle and organelle transport along microtubules to and from the periphery; and seven proteins decrease in T2D (FUBP3, TPPP, NDUFAB1, ATP2B4, NPLOC4, DHRS4, CHMP3; ROC AUC = 0.81, 0.81, 0.77, 0.83, 0.82, 0.83, 0.76) revealing disruption in gene transcription, microtubule stability, mitochondrial function, Ca^2+^ signalling, unfolded protein response, redox status and multivesicular body formation. Two secreted proteins (APOA4 and RBP4; ROC AUC = 0.79 and 0.80) were also increased in T2D. Modelling using k-fold cross-validation, identified predictive power for 17 of the 23 proteins. At the mRNA level only borderline significant changes in *Chmp3* and *Fubp3* were detected using the duodenum of the pre-clinical high-fat diet C57BL/6J mouse model of pre-diabetes.

**Conclusion:**

Overall, this study addresses a critical knowledge gap, supports our hypothesis, and contributes to a deeper understanding of intestinal involvement in T2D pathogenesis, by examining how changes in the duodenum proteome landscape might reveal mechanisms that contribute to metabolic imbalance in T2D. While these results are currently purely cross-sectional and associative, and their contribution to causality and pathophysiological interpretation for T2D remain to be determined, they expand the frontiers of research on gut-targeted therapies in T2D, and lay groundwork for future translational research into gut-centred drug development to treat T2D or achieve controlled prevention or medical remission of T2D.

**Supplementary Information:**

The online version contains supplementary material available at 10.1186/s12014-026-09595-3.

## Background

Worldwide circa 589 million adults (20–79 years) have diabetes with prevalence predicted to increase 46% by 2050. Overall, 90–95% of cases are type 2 diabetes (T2D) [1]. The development and progression of T2D is intimately linked to lifestyle choices such as diet, exercise and stress, and may be largely preventable and potentially reversible [2–4]. However, in spite of lifestyle changes and intense pharmacological intervention on glycaemia, there is relentless loss of metabolic control over time and serious ensuing co-morbidities including cardiovascular, neuropathic and vascular disease [5]. Therefore, new avenues of understanding on the aetiology of pre-diabetes and T2D are urgently needed to help find novel pharmacological targets and treatment approaches.

Following duodenal/jejunal bypass (e.g., Roux-en-Y gastric bypass), T2D remission is frequently observed and maintained for a remarkable period of time lasting years [6, 7]. It could be linked to changes in nutrient exposure, as re-exposure of the bypassed gut to nutrients reverses the prior observed remission [8]. The reasons behind the observed remission remain to be elucidated. This highlights the importance of investigating the proximal small intestine to understand remission mechanisms and find novel therapeutic targets for T2D.

Our hypothesis is that exclusion of duodenum/jejunum in T2D prevents release of unidentified factors that impair insulin sensitivity, leading to T2D remission. We hypothesize that, in T2D, lifestyle induced damage to the proximal small intestine could affect signalling pathways crucial for global glucose homeostasis, which, over time, precipitate the development of T2D. In this study we investigated the human duodenal tissue proteome, derived from non-diabetic, pre-diabetic and T2D surgical biopsies, to identify novel duodenal therapeutic targets and associated molecular and cellular mechanisms of T2D aetiology. We also used a well-established rodent pre-clinical model of pre-diabetes (C57BL/6J mice with 12-weeks of high-fat diet) for preliminary validation of the human proteomics targets, for further pre-clinical studies. We aim to discover novel potentially actionable therapeutic protein targets for the development of novel therapies to achieve medical prevention and remission of T2D.

## Materials and methods

### Human samples - informed consent and ethics committee approval

Human formalin fixed paraffin embedded (FFPE) duodenal tissue biopsies of healthy tissue were obtained from scheduled gastric resection surgeries, provided by the Digestive Pathology Clinic of the “Instituto Português de Oncologia do Porto FG” (Portugal). Ethical approval was obtained according to ethical principles and applicable international (EU Directive 2004/23/EC) and national law (registration no.133/2019/CEFCM). Informed consent was obtained from all participants.

### Human samples - inclusion criteria and grouping of participants

Inclusion criteria were: no type 1 diabetes and age > 18. Samples were divided into three groups: Group 1, non-diabetic (N, *n* = 11) having pre-operative and fasting glycaemia ≤ 99 mg/dl or HbA1C < 5.7; Group 2, pre-diabetic (P, *n* = 6) having pre-operative glycaemia or fasting glycaemia above 100 mg/dl or HbA1C ≥ 5.7; and Group 3 (D, *n* = 9) having diagnosed T2D. Duodenum samples from a total of 26 participants were collected from July 2020 to March 2022 and FFPE preserved.

### Mouse model C57BL/6J of high-fat diet induced pre-diabetes

Mouse duodenum tissue was obtained from C57BL/6J male mice, bred inhouse, placed on a control (*n* = 6) or a high fat diet (*n* = 6) for 12 weeks from the age of five weeks. All animal procedures were approved by the national ethical body for animal experimentation (DGAV ref 0421/000/0002022). Diet was UV treated formula (Ssniff Spezialdiaten): high-fat diet (HFD, D12451, 45 kJ% fat) and normal control diet (NCD, D12450H, 10 kJ% fat, 16% sucrose). Mice were maintained at 22° C on 12-hour light-dark cycle. Pre-diabetic model was verified at endpoint by 2-hour oral glucose tolerance test (2 g glucose/kg mouse body weight). At endpoint mice were sacrificed by cervical dislocation. Duodenum tissue collected was preserved at −80° C.

### Proteomics of human FFPE duodenum tissue by LC-MS/MS and global data processing

Proteins were extracted for mass spectrometry, from six 10 μm thick sections of 26 FFPE human duodenum tissue samples, using Qproteome^®^ FFPE Tissue Kit (Qiagen) following manufacturer instructions. After trypsin digestion and desalting, peptides were quantified by fluorescence. Label free quantitation (LFQ) of peptides was performed for all samples in a single batch at i3S Proteomics platform (University of Porto, Portugal) as previously described [9]. Briefly, 500 ng of peptides from each sample were analysed on a nano LC-MS/MS system with Ultimate 3000 liquid chromatography system coupled to a Q-Exactive Hybrid Quadrupole-Orbitrap mass spectrometer (Thermo Scientific, Bremen, Germany). Positive data-dependent acquisition was controlled by Xcalibur 4.0 and Tune 2.9 software using a full scan (m/z380-1580) and subsequent HCD MS/MS detection of the 10 most intense peaks from a full scan. Raw peptidomics data was analysed using MaxQuant software (v2.6.3, against *Homo sapiens* reference proteome (UP000005640_9606.fasta with 20,666 entries and additional isoforms P000005640_9606_additional.fasta with 84,307 entries, accessed on 26th of July 2024 https://ftp.uniprot.org/pub/databases/uniprot/current_release/knowledgebase/reference_proteomes/Eukaryota/UP000005640) integrated with Andromeda search engine [10–13]. Search parameters included variable modifications for oxidation (M), acetyl (protein N-term) and deamidation (NQ); Label Min. ratio count = 1, Min. peptide Length = 6, PSM FDR = 0.01, including razor peptides. For quantification the sum of peptide intensities for each protein normalized to the number of theoretically observable peptides was used, providing Intensity-based Absolute Quantification (iBAQ) values. MaxQuant parameters, proteinGroups and peptide files are available as supplementary material in the online version of the article (Additional Files 1, 2 and 3). Perseus software (v2.1.1.0) was used to remove contaminants and normalize data [14]. Proteins detected as potential contaminants, reverse sequences, and proteins only identified by site were removed from the data set. Data values were normalized, adjusted (sum of each column/10^11^) and Log_2_ transformed. Only proteins with at least 90% of non-missing data in at least one subject group were included. Missing values were imputed into normal data distribution of each sample.

### Additional data analysis

Linear discriminant analyses (LDA) were performed using the R statistical computing platform (R v4.4.1 and MASS v7.3–65) to verify classification of disease groups by protein levels [15]. Ranking of proteins was done by verifying all possible combinations of the minimal number of proteins having LDA confusion matrix accuracy of the model equal to one and verifying bi-plot group overlaps. Pearson correlation was used to identify correlating abundances between pairs of proteins. Receiver operating characteristic (ROC) analyses were used to evaluate performance of individual proteins in discriminating disease states. Repeated (3 repeats) 4-fold cross-validation analyses (using R package caret v7.0–1), trained, using a multinomial logistic regression model, on 70% and tested on 30% of the data, for the 23 proteins, using ten different seeds for stratified data partitioning across the three disease states, was used to evaluate predictive power of individual and protein pair combinations. The objective of these analyses was exploratory: to reduce dimensionality and find a minimal set of proteins that can differentiate the disease states; and to rank the proteins in order of contribution towards differentiating the disease states.

### In silico functional annotation of proteins

Proteins functions and localization were examined using searches in PubMed (https://pubmed.ncbi.nlm.nih.gov/), Human Protein Atlas (https://www.proteinatlas.org/), Database for Annotation, Visualization and Integrated Discovery (https://david.ncifcrf.gov/, v2021 DAVID Knowledgebase v2023q4) and UniProt (https://www.uniprot.org/, Release 2024_01) [16, 17]. 

### RNA extraction and relative mRNA expression

Total RNA was extracted from 6 to 8 10 μm sections of each FFPE sample using E Z N A ^®^ FFPE RNA Kit (Omega Bio-tek, Cat. no. 323R6954-01). Total RNA was isolated from frozen mouse duodenum tissue using a Dounce tissue grinder with phenol-guanidine-isothiocyanate buffer (38% phenol (Fisher Chemical P/2360/5), 0.8 M guanidine thiocyanate (Fisher Bioreagents BP221-1), 0.4 M ammonium thiocyanate (Labkem AMTH-00 A-500), 0.1 M sodium acetate (EPR SOAC-A0P-1K0), 5% glycerol (Fisher Chemical G/P450/08)), chloroform (Alfa Aesar L14759-AP) organic extraction, and isopropanol (Fisher Bioreagents BP2618-1) precipitation [18]. RNA concentration was determined on a Nanodrop 2000 (Thermo Fisher Scientific). Relative gene expression was determined by two step Reverse Transcription Real Time Quantitative PCR with validated reference genes [19]. cDNA was prepared from 2 µg of total RNA with 1 st Strand cDNA Synthesis Kit (EntiLink™ Eq. 003) with random primers N6 and Oligo (dT)18 in 20 µl reaction volume using PCR Biorad MyCycler (10 min 25° C, 90 min 42° C, 5 min 85° C). mRNA gene expression of two technical replicates was determined from 100 ng cDNA, gene specific primer pairs and 2x NZYSupreme qPCR Green Master Mix (NZYTech MB44103) in a 10 µl reaction volume using Rotor-Gene Q (v2.3.4, QIAGEN): 2 min 95° C; and 40 cycles of 15 s 95° C, 30 s 57° C and 30 s 72° C). Relative mRNA expression was determined by the 2^− ΔΔCt^ method using *B2M* or *Actb* genes as reference. Forward and reverse primers (Table 1) for human *CHMP3*, *FUBP3*, *KIF5B*, *DHRS4*, *NDUFAB1*, *B2M* and mouse *Chmp3*, *Fubp3*, *Kif5b*, *Dhrs4*, *Ndufab1*, *Actb* gene expression were designed across exon junctions using Primer Quest (Integrated DNA Technologies, IDT).

**Table 1:** Primers used for real-time RT-qPCR

Gene	Species	Forward primer sequence (5'−3')	Reverse primer sequence (5'−3')
CHMP3	Hs	GATGAAGGCTGGGATCATAGAG	TCTGCTTCTTCCTCCATTTCTT
FUBP3	Hs	CTTACAGCCCAGGAAAGAGAC	CTATGACGAGGCCACACTTATC
KIF5B	Hs	GCAAGCAGTTAGAAAGCACAC	GGCTTCATGTTGAGAGATACGA
DHRS4	Hs	CCCTGCGGATAAGAAGGTTAG	ACCCACCACCACTGTTTC
NDUFAB1	Hs	ACCCAGAGAACTTTCAGTAAAT	TTTCAAACCCAAATTCGTCTTCC
B2M	Hs	CTATCCAGCGTACTCCAAAG	GGATGGATGAAACCCAGAC
Chmp3	Mm	CTGCAGAGAGCACAGAAGT	TGATTCCAGCCTTCATCATCTC
Fubp3	Mm	GGAGGTAGTATAGAGGTGTCTGT	GCGTCGTTCTGGATCTTCTT
Kijf5b	Mm	CTTCGGATCTCCCAACATGAA	CCTCACCAAGGGAATCAACA
Dhrs4	Mm	CCATGCAAATCAGAAGGCTAGG	CCACTACTACGGTCTCTCCATT
Ndufab1	Mm	GGACCGAGTTCTGTATGTCTTG	TTTCAAACCCAAATTCGTCTTCC
Actb	Mm	CAGCCTTCCTTCTTGGGTATG	GGCATAGAGGTCTTTACGGATG

Hs- Homo sapiens, Mm- Mus musculus

### Statistical analysis

Statistical analyses were performed using the R statistical computing platform (R v4.4.1) and packages tidyverse v2.0.0, dlookr v0.6.3, ggplot2 v3.5.1, plotly v4.10.4, ggstatsplot v0.13.0, car v3.1–3, psych v2.4.12 and dplyr v1.1.4.^15^ Normality and homogeneity of variance were determined using Shapiro-Wilk normality test and Levene’s Test. Significance of differences in protein levels or relative mRNA expression was analysed using Fischer’s one way Analysis of Variance (ANOVA), Welsh’s ANOVA or Kruskal-Wallis rank sum test. Student’s t test, Games-Howell test or Dunn test were used for pairwise comparisons. In pairwise comparisons Holm adjusted p-values were determined across disease groups. Significance threshold level of 5% was used for all statistical tests: p-values are indicated by value or * < 0.05, ** < 0.01, *** < 0.001, **** < 0.0001 and ***** < 0.00001.

## Results

### Study population characteristics

**Fig. 1:**
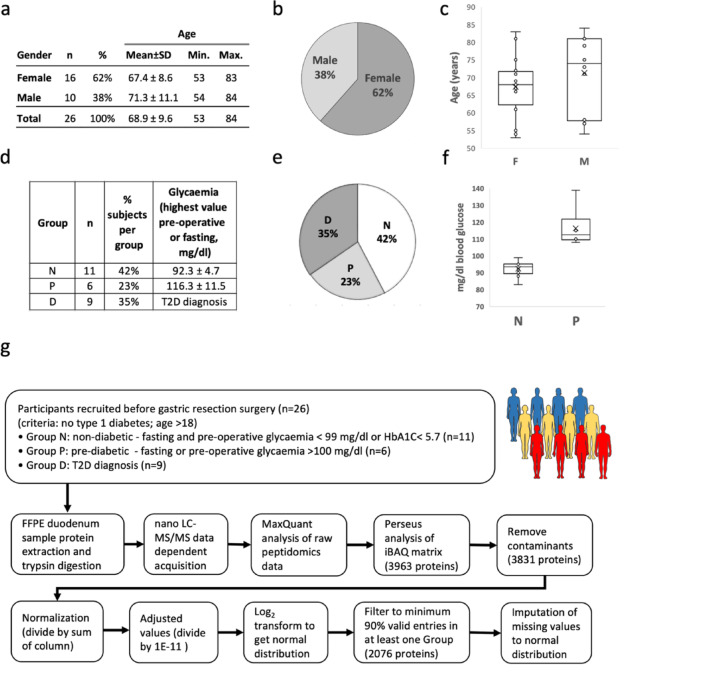
Patient groupings and proteomics workflow. **(a)** Age and gender variables; **(b)** Gender category distribution; **(c)** Age distribution; **(d)** Grouping according to T2D diagnosis or highest value of pre-operative or fasting glycaemia; **(e)** Group category distribution; **(f)** Glycaemia distribution for non-diabetic and pre-diabetic subjects (one subject with missing pre-operative and fasting glycaemia was classified as non-diabetic with HbA1c < 5.7%. **(g)** Flow chart overview of samples, proteomics workflow, peptide analysis, inferred proteins and data processing before statistical analysis. N – no diabetes, P – pre-diabetes, D – type 2 diabetes

Participants included 16 females (62%) aged 53–83 (mean age 64.7 ± 8.6) and 10 males (38%) aged 54 to 84 (mean age 71.3 ± 11.1) (Fig. 1a, b and c). Subject grouping according to T2D diagnosis or glycaemia provided eleven non-diabetics (42%, N), six pre-diabetics (23%, P) and nine were diagnosed type 2 diabetics (35%, D) (Fig. 1d and e). Average glycaemia of non-diabetics was 92.3 ± 4.7 mg/dl, and of prediabetics was 116.3 ± 11.5 mg/dl (Fig. 1f).

### Proteomics of human pre-diabetic and T2D duodenum by LC-MS/MS

**Fig. 2:**
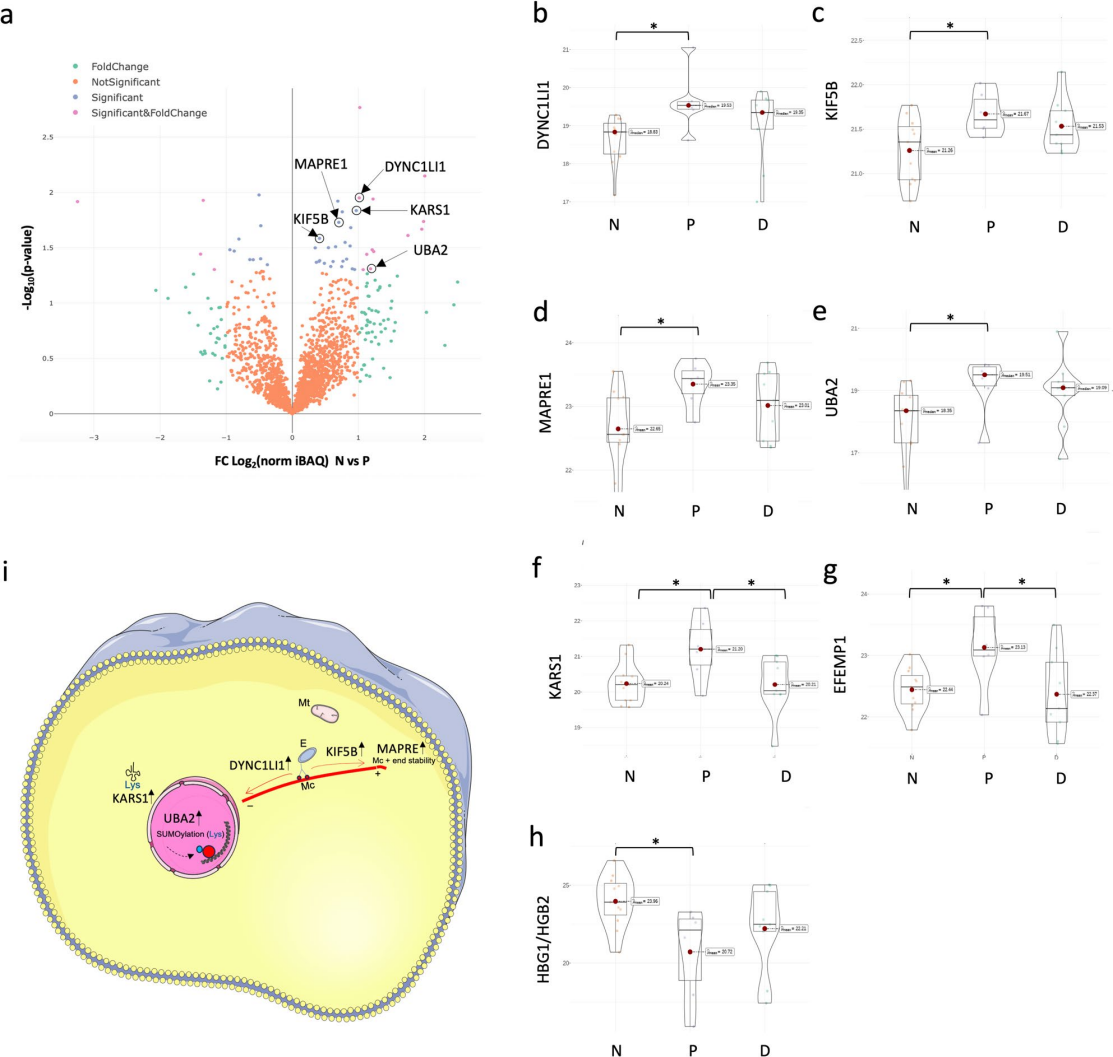
Differential levels of LC-MS/MS proteome data in pre-diabetes in human duodenum. **(a)** Volcano plot of 2076 protein level pairwise comparisons between non-diabetic versus pre-diabetic samples (non-adjusted p-values). **(b-h)**. Violin plots of all proteins with significant differences in prediabetes (one-way ANOVA *p* > 0.05). Significant Holm adjusted p-values for pairwise comparisons displayed (*) *p* > 0.05. **(i)** Illustration depicting function and localization of intracellular proteins (image composed with support from Servier Medical Art, https://smart.servier.com/). FC - fold change, N - non-diabetic, P - pre-diabetic, D - type 2 diabetic. Significant and Log_2_ fold change > 1 or <−1 are colour coded. The position of the top-ranking proteins is indicated by arrows on the plots. Volcano plots were generated with R package plotly v4.10.4. Mc - microtubule, Mt - mitochondria, E - endosome, Lys - lysine

**Fig. 3:**
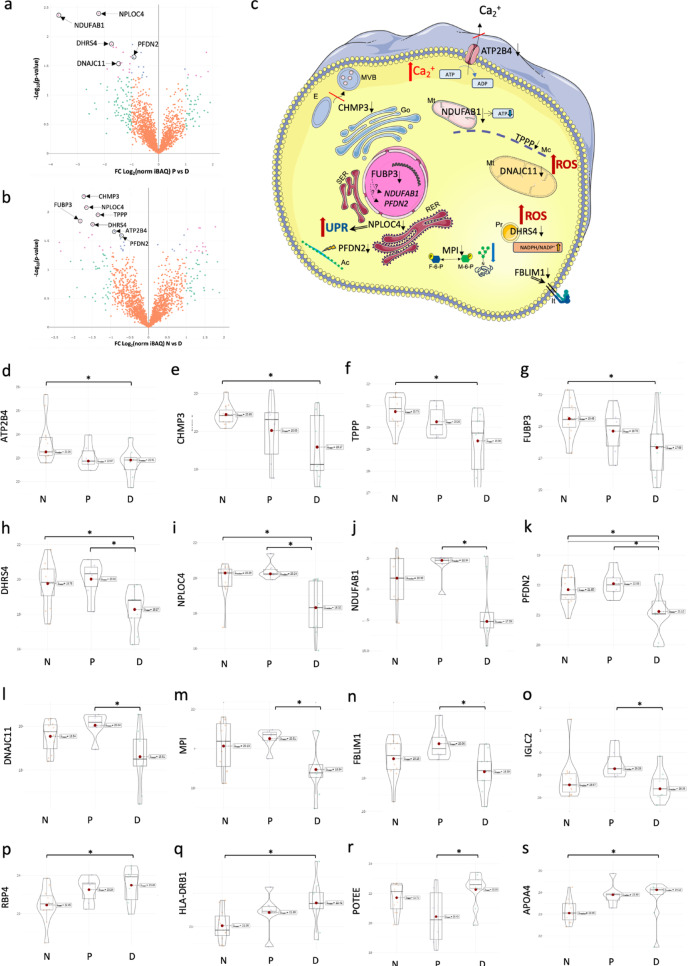
Differential levels of LC-MS/MS proteome data in T2D in human duodenum. **(a**,** b)** Volcano plots of 2076 protein level pairwise comparisons between pre-diabetic versus type 2 diabetic, and non-diabetic versus type 2 diabetic samples (non-adjusted p-values). **(c)** Illustration depicting function and localization of intracellular proteins (image composed with support from Servier Medical Art, https://smart.servier.com/). **(d-s)** Violin plots of all proteins with significant differences in type 2 diabetes (one-way ANOVA *p* > 0.05). Significant Holm adjusted p-values for pairwise comparisons displayed (*) *p* > 0.05. FC - fold change, N - non-diabetic, P - pre-diabetic, D - type 2 diabetic. Significant and Log_2_ fold change > 1 or < −1 are colour coded. The position of the top-ranking proteins is indicated by arrows on the plots. Volcano plots were generated with R package plotly v4.10.4. Mc - microtubule, Mt - mitochondria, E - endosome, MVB - multi vesicular body, Pr - peroxisome, It - integrin, Ac - actin, RER - rough endoplasmic reticulum, SER - smooth endoplasmic reticulum, Go - Golgi apparatus, UPR - unfolded protein response, F-6-P - fructose-6-phosphate, M-6-P - manose-6-phosphate

Using MaxQuant analysis of peptidomics LC-MS/MS peaks of 26 human duodenum samples from non-diabetic, pre-diabetic and T2D samples, we initially identified and quantified 3963 proteins. After removal of potential contaminants, iBAQ data was normalized, Log2 transformed and filtered to include only proteins with 90% valid values in at least one group (N, P or D). Missing data was imputed to global normal data distribution within each sample using Perseus software (Fig. S1) before statistical analysis of the 2076 proteins identified (Fig. 1g). One-way ANOVA analysis across disease states revealed significant changes in 23 proteins (Additional File 4) with at least one significant Holm adjusted pairwise comparison between groups (N, P or D). In proteomics data set for non-diabetic versus pre-diabetic groups (Fig. 2a), we identified increase in five intracellular proteins (DYNC1LI1, KIF5B, MAPRE1, UB2A and KARS1) (Fig. 2b-f and i) essentially involved in movement of organelle cargo to and from the cell periphery and nuclear SUMOylation, and one extracellular protein EFEMP1; and decrease in HGB1/HGB2. In T2D proteomics data (Fig. 3a-b) the abundance of 14 proteins is significantly decreased. Major function of these (Fig. 3c-s) include regulation of calcium ion removal from cytoplasm (ATP2B4); multi-vesicular-body formation (CHMP3); microtubule integrity (TPPP); steroid redox metabolism in peroxisomes (DHRS4); unfolding and extraction of ubiquitinated proteins for proteasomal degradation (NPLOC4); protein folding of actin cytoskeleton (PFDN2); mitochondrial energy delivery (NDUFAB1); mitochondrial structural integrity (DNAJC11); protein mannose glycosylation (MPI); integrin activation (FBLIM1); adaptive immune responses (IGLC2); and transcriptional regulation (FUBP3). In T2D we also observed an increase in three circulating proteins (RBP4, HLA-DRB1 and APOA4) involved in retinol transport, antigen presentation and lipoprotein functions respectively; and increase in a primate specific protein POTEE involved in cell motility.

### Linear discriminant, correlation, cross-validation and receiver operating characteristic analyses of human duodenum proteomics data

**Fig. 4:**
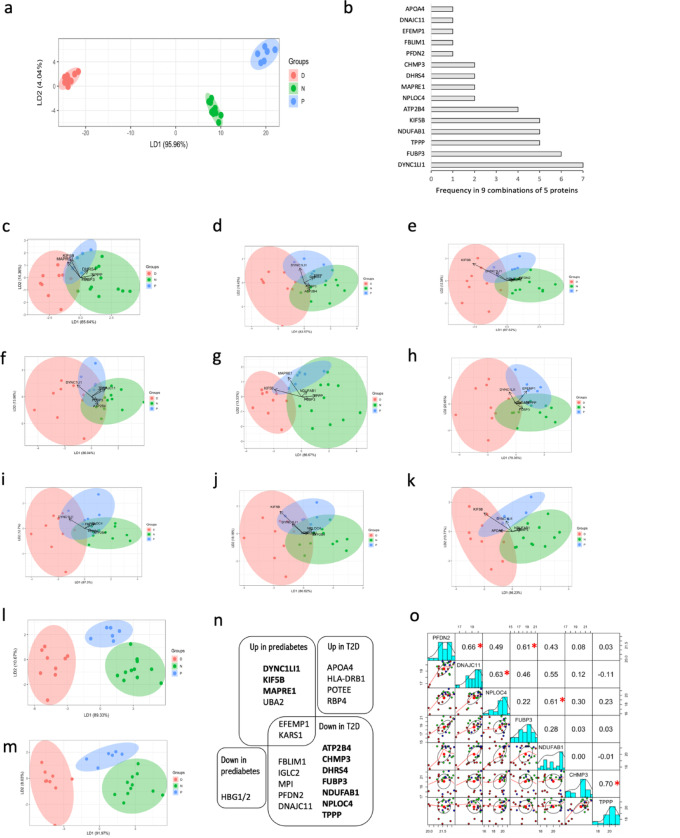
Analysis of highest-ranking proteomics targets. **(a)** LDA analysis bi-plot of all 23 proteins with significant changes identified in one-way ANOVA analysis shows clear separation of disease groups. **(b)** Ranking, by frequency of occurrence, of the 15 proteins that occur in the only 9 unique combinations of 5 proteins with LDA confusion matrix accuracy = 1. **(c-k)** LDA bi-plots of the only 9 combinations of 5 proteins with confusion matrix accuracy = 1, which are: **(c)** DHRS4 + TPPP + FUBP3 + KIF5B + MAPRE1, **(d)** DHRS4 + TPPP + FUBP3 + DYNC1LI1 + ATP2B4, **(e)** PFDN2 + CHMP3 + KIF5B + DYNC1LI1 + NDUFAB1, **(f)** TPPP + DNAJC11 + FUBP3 + DYNC1LI1 + ATP2B4, **(g)** TPPP + FUBP3 + KIF5B + MAPRE1 + NDUFAB1, **(h)** TPPP + FUBP3 + EFEMP1 + DYNC1LI1 + NDUFAB1, **(i)** FUBP3 + FBLIM1 + NPLOC4 + DYNC1LI1 + ATP2B4, **(j)** KIF5B + NPLOC4 + DYNC1LI1 + NDUFAB1 + ATP2B4, **(k)** CHMP3 + KIF5B + DYNC1LI1 + NDUFAB1 + APOA4. **(l)** LDA bi-plot of the 15 proteins. **(m)** LDA bi-plot of the 10 top ranking proteins present in at least two combinations of 5 proteins with LDA accuracy = 1. **(n)** Venn diagram summary of the 23 proteins. **(o)** Pairs panels of correlations of protein pairs; Pearson correlation coefficient above 0.60 identified by red * (full data set in Fig. S2). Diagonal panels show histograms of data from each individual protein. Lower left panels show scatter plots of data for each pair of proteins: green (non-diabetic), blue (pre-diabetic), red (T2D); upper right panels show correlation coefficients

To determine whether the 23 identified proteins could accurately discriminate non-diabetic, pre-diabetic and T2D, we performed an LDA analysis. LDA of the 23 proteins classified disease groups with 95.96% separation achieved by the first linear discriminant function and with confusion matrix accuracy of the model = 1 (Fig. 4a). LDA of all possible combinations of two, three, four and five proteins, showed that a minimum combination of five proteins was required to achieve confusion matrix accuracy = 1. Of the 33,649 possible combinations of five of the 23 significant proteins, we identified nine unique combinations of five proteins with confusion matrix accuracy = 1, with bi-plots showing some overlap of disease groups (Fig. 4c and k). The proteins were ranked by frequency of occurrence in the nine combinations of five proteins (Fig. 4b). Together the nine combinations gather 15 proteins for which LDA results in 89.33% separation achieved by the first linear discriminant function (Fig. 4l). Considering the ten proteins (DYNC1LI1, FUBP3, KIF5B, TPPP, NDUFAB1, ATP2B4, DHRS4, CHMP3, MAPRE1 and NPLOC4) that occur in more than one of the 9 combinations, we achieve 91.97% separation by the first linear discriminant function in LDA analysis, with clear separation of the T2D, pre-diabetes and non-diabetes groups (Fig. 4m). All 10 highest ranking of 23 proteins are summarised and highlighted in a Venn diagram (Fig. 4n). To determine the strength of association between levels of pairs of protein, Pearson correlations of protein pairs of all 23 proteins identified five positive correlations above 0.6, representing a strong correlation (Fig. 4o, full analysis in Fig. S2), namely: CHMP3/TPPP, PFDN2/DNAJC11, DNAJC11/NPLOC4, PFDN2/FUBP3 and NDUFAB1/NPLOC4, all of which occur in our LDA ranking analysis (Fig. 4b). To evaluate the ability of the protein levels to discriminate between non-diabetes and T2D, receiver operating characteristic (ROC) analysis was performed and the area under the curve (AUC) was used as the metric for overall performance. In analysis of the 23 proteins with significant changes (Table 2) all of the 10 top ranking proteins have 0.76 < AUC < 0.83 for decreased abundance in T2D and 0.82 < AUC < 0.89 for increased abundance in pre-diabetes. Modelling using repeated 4-fold cross-validation for all 253 pair combinations of the 23 proteins, using 10 different partitioning seeds, produced 59 pair combinations with test confusion matrix accuracy of 1, gathering 17 of the 23 proteins (R code in Additional File 5). The proteins with the highest contribution and frequency of occurrence in the pairs were DNAJC11 (12 out 59) and NPLOC4 (12 out of 59).

**Table 2:** Receiver Operating Characteristic (ROC) analysis for 23 proteins with significantchanges. AUC - area under the curve

Portein	N versus D		N versus P	
	Accuracy	AUC	Accuracy	AUC
APOA4	0.85	0.79	0.82	0.85
ATP2B4	0.85	0.83	0.82	0.76
CHMP3	0.85	0.76	0.76	0.58
DHRS4	0.85	0.83	0.65	0.56
DNAJC11	0.75	0.73	0.82	0.76
DYNC1LI1	0.85	0.75	0.94	0.89
EFEMP1	0.70	0.58	0.88	0.82
FBLIM1	0.70	0.69	0.65	0.70
FUBP3	0.80	0.81	0.65	0.65
HBG1_HBG2	0.70	0.70	0.82	0.86
HLA_DRB1	0.80	0.78	0.76	0.70
IGLC2_IGLC6	0.60	0.63	0.76	0.82
KARS1	060	0.47	0.82	0.83
KIF5B	0.70	0.68	0.76	0.82
MAPRE1	0.70	0.63	0.76	0.86
MPI	0.75	0.76	0.59	0.52
NDUFAB1	0.80	0.77	0.71	0.67
NPLOC4	0.80	0.82	0.59	0.41
PFDN2	0.80	0.78	0.65	0.62
POTEE	0.70	0.67	0.82	0.68
RBP4	0.80	0.80	0.82	0.79
TPPP	0.75	0.81	0.76	0.71
UBA2	0.75	0.73	0.88	0.85

### mRNA expression of equivalent proteomics targets in duodenum of human and pre-clinical mouse model of pre-diabetes

**Fig. 5:**
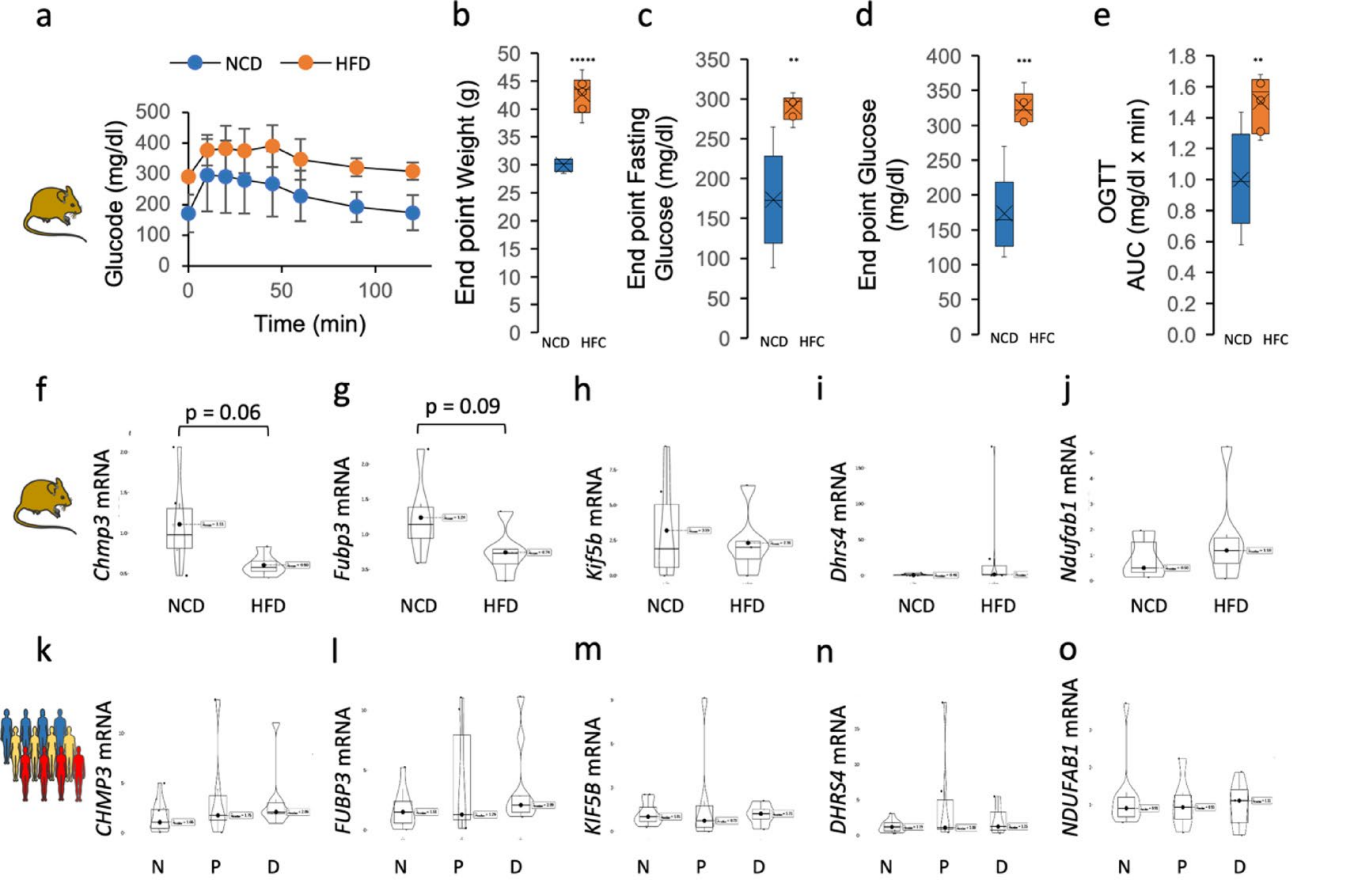
Quantitative real-time RT-PCR of five selected proteins in mouse T2D model (12-week high-fat diet C57BL/6J mouse model of prediabetes (*n* = 6)). **(a)** 2 h glycaemia variation in OGTT. **(b)** Weight of mice at end-point. **(c)** End point fasting glycaemia before OGTT. **(d)** End point glycaemia after OGTT. **(e)** OGTT area under the curve (AUC). **(f-j)** Relative mRNA expression (quantitative real-time RT-PCR) of *Chmp3*, *Fubp3*, *Kif5b*, *Dhrs4*, *Ndufab1* in NCD and HFD mouse duodenum. **(k-o)** Relative mRNA expression (quantitative real-time RT-PCR) of *CHMP3*, *FUBP3*, *KIF5B*, *DHRS4*, *NDUFAB1* in human tissue from FFPE samples used in proteomics analysis. NCD – normal control diet, HFD – high-fat diet, OGTT – oral glucose tolerance test. N - non-diabetic, P - pre-diabetic, D - type 2 diabetic. Student t-test p-values for 5% significance level: ** < 0.01, *** < 0.001 and ***** < 0.00001

To examine the direction of change, at gene expression level, of equivalent proteins in the mouse pre-diabetic model (Fig. 5a and e), relative mRNA expression of five mouse genes *Chmp3*, *Fubp3*, *Kif5b*, *Dhrs4* and *Ndufab1* (Fig. 5f and j) were determined in mouse duodenum. Borderline significant decrease in relative mRNA expression was observed for *Chmp3* and *Fubp3*. Relative mRNA expression of five corresponding genes (*CHMP3*, *FUBP3*, *KIF5B*, *DHRS4* and *NDUFAB1*, Fig. 5k-o) in human duodenum was also evaluated with no significant changes observed.

## Discussion

This study addressed a critical knowledge gap examining how changes in the duodenum proteome landscape might reveal mechanisms that contribute to metabolic imbalance in T2D. Significant changes were observed in prediabetes and even more so in T2D (Figs. 2 and 3). In pre-diabetes increase in three microtubule related proteins KIF5B, MAPRE1 and DYNC1LI1, essentially involved in movement of organelle cargo to and from the cell periphery. KIF5B, a ubiquitous motor protein critical for mitochondrial dynamics, mitochondrial transport along microtubules, ensuring proper distribution and provision of ATP supply to high energy demand sites or mitophagy, is glucose sensitive and also functions in exocytosis [20]. An increase in KIF5B in the duodenum could have implications for hyperactivation of enteric neuro-endocrine function. Lack of change observed for *Kif5b* mRNA in the mouse pre-diabetic model suggests alterations in protein turnover rather than transcription. MAPRE1 also associates to growing ends of microtubules, preventing sudden depolymerization, regulating cargo transport and cytoskeletal remodelling. DYNC1LI1, a minus-end-directed microtubule motor, is essential for selective regulated cargo transport of endosomes, lysosomes and Golgi vesicle towards the cell centre. Increase in these proteins is evidence of increased intracellular cargo dynamics, the significance of which remains to be elucidated. Increase in UB2A, a nuclear protein responsible for SUMOylation of transcription factors could be a central regulator characterising the pre-diabetic state [21]. 

The majority of changes were decreased protein abundance observe in T2D, potentially affecting multiple cellular processes, from maintenance of intracellular calcium ion homeostasis, ATP provision, mitochondrial stability, redox status, integrin activation, glycosylation, protein folding and proteo-stasis, multivesicular body formation and transcription. FUBP3 could be a possible central coordinator of changes, since it is involved both in DNA binding and transcriptional regulation (binding single-stranded DNA elements, activating transcription upstream or within transcription driven by RNA polymerase II) and RNA binding and post‑transcriptional regulation (modulating mRNA stability and translation) [22, 23]. FUBP3 has been linked to inflammatory pathways where suppressed FUBP3 was shown to alter expression of genes linked to T cell activation and inflammation, thus a decrease in FUBP3 in the duodenum might have important consequences on the regulation of local inflammation linked to food exposure in the duodenum [22, 24]. In addition we identified in published RIP-Seq data evidence for direct binding of FUBP3 to NDUFAB1 and PFDN2, two proteins identified as decreased in our data [24]. The potential role of FUBP3 on expression of these in the context of T2D duodenum alterations and possible links to glycaemic control will be pursued.

The multivesicular body (MVB) sorting pathway should also be pursued since it points to changes in secreted entities that could potentially communicate with pancreas and affect insulin secretion, or affect receptor recycling and thus affect inter-organ communication. CHMP3, a ubiquitous core component of the ESCRT‑III (Endosomal Sorting Complex Required for Transport III) complex assembles on endosomes to execute membrane scission of intraluminal vesicles [25]. It is essential for the MVB sorting pathway, that delivers transmembrane proteins to lysosomes for degradation [26]. Since CHMP3 regulates receptor trafficking and turnover, its decrease could alter membrane receptor recycling or degradation. ESCRT components also participate in autophagy and lysosomal function and exosome secretion [27, 28]. CHMP3 deficiency in the duodenum would disrupt local endo-lysosomal trafficking, membrane repair, and autophagy, all of which are critical for intestinal epithelial homeostasis, immune regulation, barrier integrity and inter-organ communication. With lower CHMP3, overactive receptor signalling may lead to chronic inflammation, that could impact local endocrine signalling relevant to glucose homeostasis. The ESCRT system also regulates exosome release, MHC-II trafficking, and cytokine secretion. CHMP3 deficiency could impair antigen presentation and communication between enterocytes, enteric neuronal and endocrine cells, and immune cells resulting in impaired tolerance to commensals. ESCRT proteins are also known to modulate Wnt signalling, crucial for intestinal stem cell proliferation. Disrupted Wnt receptor recycling due to CHMP3 loss could impair epithelial regeneration. CHMP3 is also directly involved in the biogenesis and release of extracellular exosomes. Thus, impaired CHMP3 function may reduce exosome release, potentially impacting circulating exosomes that can carry microRNAs relevant to glucose homeostasis. Indeed, low level of microRNA miR-122 is a circulating biomarker of obesity/metabolic syndrome and miR-122-5p directly targets CHMP3 3’UTR, a regulatory loop worth investigating in T2D enteric context, given the importance of exosomes in T2D [29–31]. The mouse model of pre-diabetes provided a pilot validation tool and source of high-quality duodenal RNA. The decreased mRNA expression of *Chmp3* and *Fubp3* in mouse duodenum, presented a large effect size matching the direction of change observed in the orthologous human proteins, in spite of borderline significance. Further research is being pursued to determine if CHMP3 and FUBP3 could be novel intestinal protein or gene expression targets for follow-up investigation in causality of T2D.

Disrupted mitochondrial function and redox status may also be important. NDUFAB1, a mitochondrial acyl carrier protein, operating within the inner mitochondrial membrane as an accessory subunit of complex I in the mitochondrial electron transport chain, also regulates iron–sulphur (FeS) cluster biogenesis, assists in lipoylation of key metabolic enzymes, and supports assembly of respiratory complexes and super-complexes [32, 33]. It binds a 4’-phosphopantetheine prosthetic group, which enables it to carry acyl chains during lipid biosynthesis in mitochondria. Without NDUFAB1, complex I becomes unstable or dysfunctional, which impairs oxidative phosphorylation, reduces ATP generation and increases mitochondrial ROS. DNAJC11 is also a mitochondrial protein that plays a key role in maintaining the organization of the inner mitochondrial membrane. A decrease of DNAJC11 would disrupt cristae architecture, leading to mitochondrial dysfunction [34]. Decrease in TPPP could also destabilized microtubules and impair cargo trafficking, including mitochondria. TPPP also plays an important role in microtubule dynamics, cellular stress and neurodegeneration [35]. Also implicated in redox status is DHRS4, an NADP(H)-dependent oxidoreductase that is localized primarily to peroxisomes and possibly mitochondria, which participates in steroid hormone inactivation and converts reactive α-dicarbonyls and aromatic ketones into less reactive compounds; a decrease would lower detoxification function and expose cells to oxidative and electrophilic stress [36, 37]. The ATP dependent high-affinity Ca²⁺ efflux pump, ATP2B4, is located on the basolateral membrane of intestinal epithelial cells. It maintains low cytosolic Ca²⁺ levels powered by ATP; in addition to lower protein levels, lower ATP levels resulting from dis-functional mitochondria would further impair Ca²⁺ efflux. It is also expressed in immune cells such as macrophages, which are active in gut immune surveillance, and can influence NF-κB signalling, cytokine production and immune cell activation [38]. ATP2B4 decrease would disrupt intracellular calcium balance and affect epithelial barrier stability.

In T2D, dynamics of protein folding and proteo-stasis may also be affected. PFDN2, is a subunit of the prefoldin chaperone complex that binds nascent polypeptides and delivers them to chaperonin TRiC/CCT, which completes folding. Decrease in PFDN2 could lead to protein misfolding, protein aggregation, activation of unfolded protein responses (UPR), and disrupted cytoskeleton, impairing cell division, migration, and structural integrity. DNAJC11 is part of the Hsp40/DnaJ family of molecular chaperones involved in organizing mitochondrial cristae. Disruption of DNAJC11 can thus compromise mitochondrial functions. NPLOC4, on the other hand, is not a classical chaperone, but rather part of the VCP–NPLOC4–UFD1 complex, involved in the unfolding and extraction of ubiquitinated proteins from complexes for proteasomal degradation. A failure of chaperone and NPLOC4 systems would lead to toxic protein accumulation. NPLOC4 and DNAJC11 are highlighted in our correlation, cross-validation and LDA analyses, and levels are both decreased, indicating a possible disruption in T2D functionally converging on proteo-stasis.

An increase in three secreted proteins was also observed. Increased APOA4 in T2D could be protective; primarily synthesized by enterocytes for assembly of chylomicrons carrying dietary fat, it also acts in satiety signalling, regulation of oxidative stress and inhibition of macrophage activation, helping to maintain gut barrier integrity. RBP4, on the other hand is primarily produced by the liver and adipose tissue, and circulates systemically as the primary circulating transporter of retinol to peripheral tissues. Moderate RBP4 increase could enhance retinol delivery. However, it’s increased detection in the duodenum could be associated with inflammation. It is associated with increased pro-inflammatory cytokines (e.g., TNF-α, IL-6), activation of macrophages and dendritic cells, and disruption of insulin signalling via TLR4/NF-κB pathways, that may promote low-grade inflammation in the gut and disrupt immune tolerance to microbiota [39, 40]. Finally, HLA-DRB1 forms part of the MHC class II complex, that presents extracellular antigens to CD4 + T helper cells, triggering adaptive immune responses. Excessive HLA-DRB1 may contribute to loss of immune tolerance, leading to inappropriate T cell activation against gut antigens or microbiota. The intestine is the largest neuroendocrine organ in the body, tightly integrated with the enteric immune system that interfaces with the environment; chronic changes in adaptive immune responses could affect both neural and endocrine enteric circuits and impact glucose homeostasis.

Regarding the overall interpretation of the data and analyses, several important points must be taken into consideration. The nature of the human duodenal protein extracts from FFPE samples used for the LC-MS/MS analyses has known caveats: although we enabled deamidation (N/Q) as a variable modification in MaxQuant, to account for the formalin-induced chemical changes of asparagine to aspartate and glutamine to glutamate mass shifts, we acknowledge that FFPE processing also introduces protein crosslinking and recovery biases that can affect LC-MS/MS quantitative accuracy, particularly for low-abundance proteins, and the proteomes we detected will reflect these biases. The limited sample size also restricts statistical power and model generalizability, particularly for multivariate analyses such as LDA, which can result in overfitting. The higher contribution of a particular protein (in the cross-validation analyses) or set of proteins (in the LDA analyses), to differentiate the disease states, in this particular set of data, does not necessarily imply higher causality for pathophysiological interpretation of T2D, because what we observe in these proteomes, in addition to being associative, could be a consequence of the disease state rather than a cause.

In the five cases of corresponding mRNA expression changes (CHMP3, FUBP3, KIF5B, DHRS4 and NDUFAB1) that we selected to validate, in both the human FFPE samples and the mouse pre-diabetic model, we observed either no significant changes or only borderline effects in Chmp3 and Fubp3 in the mouse model. While such discordance is common, we add that, given the predicted disruption to proteo-stasis reflected in lower levels of PFDN2 and NPLOC4, we could venture to interpret that the protein levels observed in the proteomics data are not reflected in corresponding gene transcription and translation changes, because the predicted increase in unfolded protein response (UPR) can lead to regulated degradation of selected proteins.

Although the strength of the evidence we present is modest due to the sample size, in particular for pre-diabetes (*n* = 6), the results for pre-diabetes show a clearer picture, in terms of convergence of function, than in T2D, where we observed changes in proteins involved in a wider variety of subcellular functions. This may reflect a cascade of consequences resulting from disease progression. The proteomics analysis was performed on bulk duodenal tissue, which comprises multiple cell types, including absorptive epithelial cells, enteroendocrine cells, immune cells, enteric neurons and muscle layers. Although several of the detected proteins are ubiquitously expressed (e.g., CHMP3, ATP2B4, FUBP3), others would belong to specific cellular compartments, such as HLA-DRB1. Further future work on pre-clinical validation, considering the variety of duodenal cell layers, is necessary to elaborate disease models that can be used to establish the potential of the proposed therapeutic target proteins discovered.

## Conclusions

Overall, our data support our hypothesis that in pre-diabetes and T2D the duodenum presents altered protein profiles, which suggest an intracellular duodenal T2D aetiology involving disrupted proteo-stasis and vesicular dynamics, as well as disrupted regulation of inflammation and redox status. These results remain associative and further investigation into the individual and overall role of the altered protein duodenal profiles discovered here will be necessary to demonstrate causality and expand the frontiers of research on gut-targeted therapies in T2D. This work provides preliminary groundwork for deciphering the duodenal aetiology of T2D and pushing for future research on novel gut-centric drug development for novel therapeutic targets with the aim of achieving medical prevention or remission of T2D.

## Supplementary Information

Supplementary Material 1

Supplementary Material 2

Supplementary Material 3

Supplementary Material 4

Supplementary Material 5

## Data Availability

All data supporting the findings of this study are available within the paper and its Supplementary Information.

## Abbreviations

APOA4: Apolipoprotein A4
ATP2B4: ATPase Plasma Membrane Ca2 + Transporting 4
CHMP3: Charged Multivesicular Body Protein 3
DHRS4: Dehydrogenase/Reductase 4
DNAJC11: DnaJ Heat Shock Protein Family (Hsp40) Member C11
DYNC1LI1: Dynein Cytoplasmic 1 Light Intermediate Chain 1
FBLIM1: Filamin Binding LIM Protein 1
EFEMP1: EGF Containing Fibulin Extracellular Matrix Protein 1
FFPE: Formalin fixed paraffin embedded
ES: Effect Size
FUBP3: Far Upstream Element Binding Protein 3
HFD: High-Fat Diet
HLA-DRB1: Major Histocompatibility Complex, Class II, DR Beta 1
iBAQ: Intensity-Based Absolute Quantification
IGLC2: Immunoglobulin Lambda Constant 2
KARS1: Lysyl-TRNA Synthetase 1
KIF5B: Kinesin Family Member 5B
LDA: Linear Discriminant Analysis
LFQ: Label free quantitation
MAPRE1: Microtubule Associated Protein RP/EB Family Member 1
MPI: Mannose Phosphate Isomerase
NCD: Normal Control Diet
NDUFAB1: NADH: Ubiquinone Oxidoreductase Subunit AB1
NPLOC4: NPL4 Homolog, Ubiquitin Recognition Factor
PFDN2: Prefoldin Subunit 2
POTEE: POTE Ankyrin Domain Family Member E
RBP4: Retinol Binding Protein 4
ROC: Receiver Operating Characteristic
T2D: Type 2 diabetes
TPPP: Tubulin Polymerization Promoting Protein
UBA2: Ubiquitin Like Modifier Activating Enzyme 2
