# Supplementary material for: Proteomics of human duodenum in pre-diabetes and type 2 diabetes reveals potential novel therapeutic targets for aetiology and therapeutics
Source: Clin Proteomics. 2026 Mar 16;23:24. doi: 10.1186/s12014-026-09595-3 (PMC13104399; doi:10.1186/s12014-026-09595-3)
Supplement: Supplementary file 5 — Supplementary Material 5 [file 12014_2026_9595_MOESM5_ESM.docx]

Additional file 5

### Partitioning and model training seeds used

## set.seed(1953)

## set.seed(222)

## set.seed(51)

## set.seed(16)

## set.seed(9)

## set.seed(531)

## set.seed(742)

## set.seed(5000)

## set.seed(87)

## set.seed(1)

### cross-validation R code used

library(caret);

df <-read_tsv(".../Additional_File_4.txt");

df <- as.data.frame(df);

df$State <- as.factor(df$State);

set.seed(1);

idx <- createDataPartition(

y = df$State,

p = 0.7,

list = FALSE

);

train_df <- df[idx, ];

test_df <- df[-idx, ];

set.seed(1)

ctrlspecs <- trainControl(

method = "repeatedcv",

number = 4,

repeats = 3,

savePredictions = "final"

);

set.seed(1);

mlogit_model <- train(

State ~ FEATURE1 + FEATURE1,

data = train_df,

method = "multinom",

metric = "Accuracy",

trControl = ctrlspecs,

);

predictions <- predict(mlogit_model, newdata=test_df);

predictions;

confusionMatrix(data=predictions, test_df$State)
